# Morphologic spectrum of glial tumors: an increased trend towards oligodendroglial tumors in Pakistan

**DOI:** 10.1186/1755-7682-7-33

**Published:** 2014-06-27

**Authors:** Atif Ali Hashmi, Naveen Faridi, Babar Malik, Muhammad Muzzammil Edhi, Amna Khurshid, Mehmood Khan

**Affiliations:** 1 Department of Histopathology, Liaquat National Hospital and Medical College, Karachi, Pakistan; 2 Department of Histopathology, Liaquat National Hospital and Medical College, Karachi, Pakistan; 3 Department of medical oncology, Sindh institute of urology and transplantation, Karachi, Pakistan; 4 Medical Student, Liaquat National Hospital and Medical College, Karachi, Pakistan; 5 Department of Histopathology, Liaquat National Hospital and Medical College, Karachi, Pakistan; 6 Medical Student, Dhaka Medical College, Dhaka, Bangladesh

**Keywords:** Glial tumors, Astrocytoma, Oligodendroglioma, Glioblastoma, Mixed oligoastrocytoma

## Abstract

**Background:**

Glial tumors are most common brain tumors in our population. While the exact etiology and pathogenesis is unknown, the evaluation of current trends in the frequency and morphology of glial tumors is imperative to constitute better diagnostic and treatment protocols. Data pertaining to frequency and spectrum of glial tumors is scarcely available in our population. The aim of this study was to determine the morphologic spectrum of glial tumors prevalent in our population.

**Method:**

126 cases of glial tumors were retrospectively analyzed over a period of 5 years. Patients from all age groups and both genders were included in this study. Glial tumors were classified and graded according to WHO classification.

**Results:**

Glial tumors were more common in males with a sex ratio of 2:1 and mean age of 38.26 years. Astrocytomas were most common glial tumors (51.6%) followed by oligodendrogliomas (23%). Glioblastoma was the most frequent astrocytic tumor and was incomparably frequent in older age group.

**Conclusion:**

In our study, Oligodendrogliomas and mixed oligoastrocytomas represent major pattern of disease in comparison with available regional data. Knowledge of these changing trends and patterns of glial tumor morphology and frequency can help in improvements and applications of newly emerging diagnostic and treatment modalities.

## Introduction

International data depict that tumors of central nervous system account for 1–2% of all neoplasms [1]. Brain tumors are far more common than spinal cord tumors [2]. In western population metastatic tumors to the brain precedes primary brain tumors [3,4] however this finding contradict the studies done in our population where primary brain tumors were considerably in significant numbers. National data of CNS tumors of our population shows that glial tumors are more common in our region than what is seen in the western world [5-7].Although there has been progress in the evaluation of genetic pathways involved in glial tumors [8,9] however the exact etiology and risk factors involved in the pathogenesis of glial tumors remain a mystery as brain is protected from external and internal environment by skull and blood brain barrier.

Emergence of new diagnostic and therapeutic modalities and better understanding of the biology and molecular pathology of glial tumors, have made it possible to devise specific therapies for different tumor subtypes depending upon biologic aggressiveness of the tumors as directed by histological grades [10]. For the appropriate application of these latest approaches, it is pertinent to have comprehensive information about the morphologic patterns of glial tumors including the demographic and clinical profile of the patients. Such information is scarcely available in our country [9-11] . Our study mainly focuses on glial tumors, as diagnosis of glial tumors is largely based on histology with very little role of immunohistochemistry which is mainly carried out to rule out metastatic carcinoma. However in western countries a few centers routinely do molecular studies to confirm the diagnosis of oligodendroglioma which is not widely available in our country [12], therefore correct histopathological recognition of oligodendroglioma is absolutely critical in our region as these cancers are highly curable due to their property of being more radio and chemosensitive [13].

The purpose of our study is to assess the histopathologic spectrum of glial tumors in our population. This will guide the clinicians to establish measures for an improved diagnosis and management protocols, including the use of targeted chemotherapy for specific subtypes. Furthermore it will also open the door for more targeted research to determine the etiologic, molecular and genetic factors involved in the genesis of glial tumors which are common in our population.

## Methods

In this study we retrospectively analyzed, 126 cases of glial tumors from January 2008 till December 2012 who underwent craniotomy or stereotactic brain biopsy for space occupying lesions of the brain. Approval from ethical review committee of Liaquat national hospital and medical college was taken prior to the start of the study. Cases of non-glial tumors were excluded from the study. After gross examination routine histological sections were made using hematoxylin and eosin stains. Immunohistochemical stains were not performed routinely. Stains using GPAF antibody was done in cases where the diagnosis is in doubt. In cases where differential is metastatic carcinoma or meningioma stains with pan-cytokeratin and EMA were used respectively. Glial tumors were classified and graded according to WHO classification 2007. Ki67 index was performed in a few cases to aid in the determination of histological grade. The final diagnosis was made by senior histopathologists. Two pathologists with more than 5 years post fellowship experience of surgical pathology reviewed the cases. The diagnosis and grading was done according to WHO classification of CNS tumors and the results were analyzed by SPSS software version 19.

## Results

A total of 126 glial tumors were diagnosed during the study period. Patients’ mean age was 38.26 years ±17.24 (1–75 years) with most common presentation in middle age group between 18 and 50 years (64.3%), followed by greater than 50 year age group (23.6%) while only 12% of patients presented before 18 years of age. 85 cases were diagnosed in males with a M: F of 2:1.

**Figure 1:**
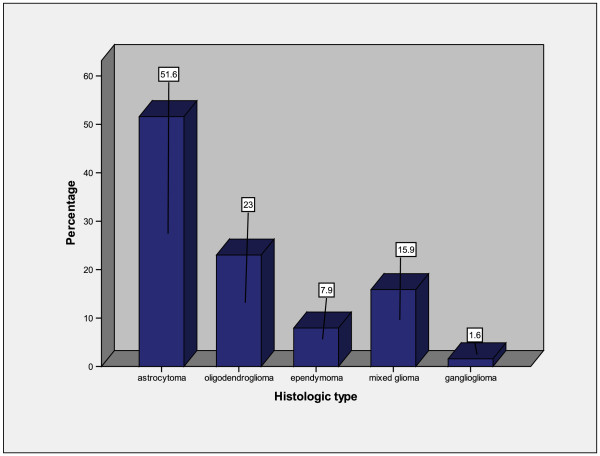
Histologic types of glioma.

Astrocytoma comprised the most common group with 65 cases (51.6%) followed by oligodendroglioma. Gangliogliomas were the least frequent category (Figure 1). Most of the tumors were grade III or IV according to WHO grading system. Overall 37% of tumors were WHO grade III, followed by grade II (26%), grade IV (24%), while grade I tumors were least common (12%). Mean age for astrocytomas and mixed gliomas were found to be higher than other tumors specifically ependymomas which were more frequent in younger population (Table 1). Gender distribution among glioma subtypes was not significantly different with a same sex ratio of 2.

**Table 1:** Age distribution of glioma subtypes

**Histologic types**	**Mean age (years)**	**Range (years)**	**Standard deviation**
**Astrocytoma**	**40.32**	**4-75**	**18.74**
**Oligodendroglioma**	**36.96**	**12-65**	**12.79**
**Ependymoma**	**28.91**	**1-65**	**16.79**
**Mixed Glioma**	**40.9**	**10-75**	**15.86**
**Ganglioglioma**	**14.5**	**7-22**	**10.61**

**Figure 2:**
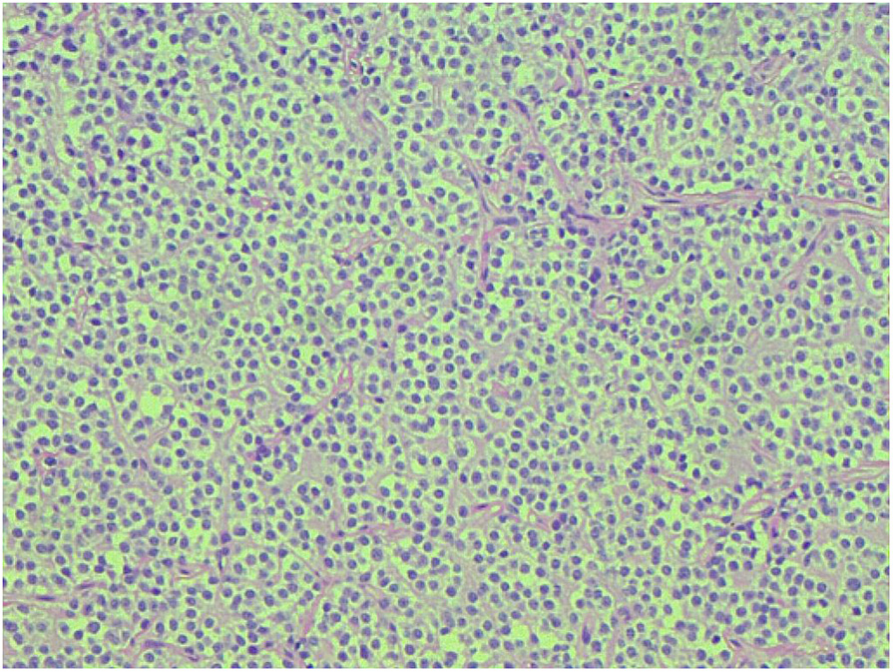
Oligodendroglioma WHO grade I, showing round nuclei with perinuclear halos and background chickenwire vasculature.

**Figure 3:**
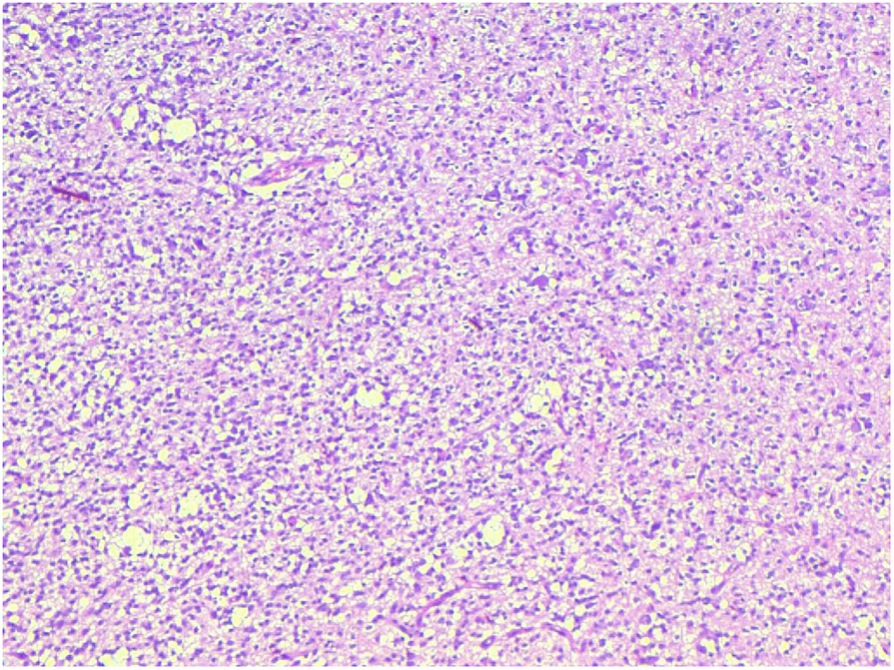
Anaplastic Oligodendroglioma, WHO grade III, showing greater nuclear atypia and mitotic activity with morphologic features compatable with Oligodendroglioma.

Among 65 cases of astrocytic tumors, glioblastoma was most frequent with a frequency of 38.5% (Table 2). Low grade circumscribed astrocytomas (pilocytic, pilomyxoid, pleomorphic xanthoastrocytoma) and diffuse astrocytoma were significantly found in younger age group as compared to high grade glioblastoma (Table 3).High grade (grade III) anaplastic oligodendrogliomas were more common (68%) than low grade (grade II) oligodendroglioma (32%) (Figures 2 and 3). The reverse is true for ependymomas where low grade (II) tumors were more common (55%) in contrast to grade III ependymomas (45%). Among mixed tumors grade III oligoastrocytomas were more frequent (75%) and there were 4 cases of glioblastoma with oligodendroglioma component (grade IV).

**Table 2:** Histologic subtypes of astrocytic tumors

**Histologic subtypes**	**WHO grade**	**Frequency**	**Percentage (%)**
**Pilocytic astrocytoma**	**I**	**11**	**16.9**
**Pilomyxoid astrocytoma**	**II**	**1**	**1.5**
**Pleomorphic xanthoastrocytoma**	**II**	**3**	**4.6**
**Subependymal giant cell astrocytoma**	**I**	**2**	**3.1**
**Diffuse astrocytoma**	**II**	**8**	**12.3**
**Gemistiocytic astrocytoma**	II/III*	**9**	**13.8**
**Gliobastoma multiforme**	**IV**	**25**	**38.5**
**Gliosarcoma**	**IV**	**2**	**3.1**
**Total**		**65**	

*4 cases of gemistiocytic astrocytoma were grade II, while 5 were grade III.

**Table 3:** Age distribution of subtypes of astrocytic tumors

**Histologic subtypes**	**Age categorIes**			**Total**	**P-Value**
	**<18 yr**	**18-50 yr**	**>50 yr**		
**Pilocytic astrocytoma**	**5**	**6**	**0**	**11**	0.0000*
**Pilomyxoid astrocytoma**	**0**	**1**	**0**	**1**	
**Pleomorphic xanthoastrocytoma**	**0**	**3**	**0**	**3**	
**Subependymal giant cell astrocytoma**	**2**	**0**	**0**	2	
**Diffuse astrocytoma**	**0**	**8**	**0**	**8**	
**Gemistiocytic astrocytoma**	**0**	**3**	**6**	**9**	
**Anaplastic astrocytoma**	**0**	**4**	**0**	**4**	
**Gliobastoma multiforme**	**2**	**10**	**13**	**25**	
**Gliosarcoma**	**0**	**0**	**2**	**2**	
**Total**	**9**	**35**	**21**	65	

*p- value is significant at <0.05 interval.

## Discussion

To our knowledge, current study is among only few studies done in our country evaluating the frequency of brain tumors and the first study specifically demonstrating the spectrum of glial tumors in our population.

CNS tumors are currently classified by histology and location within the brain. Gliomas further categorized as astrocytomas, oligodendrogliomas, and ependymomas from the cell of origin are the commonest CNS malignancies globally accounting for approximately 70% of the cases. There is a reported tendency toward a higher incidence of gliomas in highly developed, industrialized countries and some reports indicate that Caucasians are more prone than African or Asian populations [14]. Karachi South (KS) is a moderate risk population for CNS malignancies graded by the GLOBOCAN scale of I-V [15]. A study conducted at Lahore evaluated 100 cases of intracranial space occupying lesions, but the study included non-neoplastic conditions like tuberculosis, fungal infections, vascular malformations and non-neoplastic cysts [16]. We excluded all non-neoplastic conditions and specifically focused on the statistics of glial tumors. In their study neoplastic lesions comprised 89% of cases, among which glial tumors were 41%. Among total 39 glial tumors in their study, astrocytomas comprised the largest group accounting for 35 cases as reflected in our study; however they found low grade astrocytomas as more common entity than glioblastoma ,which was prevelant in our sample size. The frequency of oligodendroglioma and oligoastrocytoma were 4.8% and 2.4% respectively, far less than that observed in our study. The most common age group for CNS tumors in their study was 20–29 years with a second peak in 60–69 years. The sex ratio was 1.7: 1 correlating with the results of our study. They did not find any significant association with age groups and sex ratio, however we found glioblastoma more frequent in older population above 50 years of age.

Another study involving 386 intracranial space occupying lesions was done in Karachi [17]. Once again this study also included all non-neoplastic lesions including infections, abscesses etc. Similar to our series they reported a higher frequency of glial tumors in males with a male to female ratio of 2:1. According to their data, astrocytomas comprised around 80% and oligodendrogliomas accounted for 8.8% of all glial tumors, whereas our study depicted a higher frequency of oligodendrogliomas making up 23% of glial tumors. The frequency of mixed oligoastrocytoma was also significantly lower than that seen in our study accounting for only 2% of cases.

A third study conducted in Karachi included a total of 100 brain tumors in all age groups [18]. According to their results CNS tumors were most frequent in 5th decade followed by fourth decade. In their study gliomas comprised 58% of all brain tumors with astrocytomas accounting for the bulk of glial tumors (75%) and glioblastoma at the top of the list. Again in their series the frequency of oligodendroglioma (12.5%) and oligoastrocytoma (0.03%) was less than that seen in our study.

The largest study so far in Pakistan was done in Karachi that included 996 cases of central nervous system tumors [19]. However in this study tumors of peripheral nerves and spinal cord were also included whereas we specifically looked at glial tumors and tumors arising from nerves and meninges were excluded from our study. The glial tumors comprised 54% of cases with an age range of 1–90 years. Mean age was 43 years slightly higher than what we found. Astrocytomas were the most frequent glial tumors in their series comprising 71% of all gliomas and glioblastoma was the most common subtype(71.4%). The frequency of oligodendroglioma was 15% and that of ependymoma was 9%. Mixed gliomas comprised 3.2% of cases. In contrast the frequency of oligodendrogliomas and mixed gliomas were lower than that seen in our series.

Another study conducted in India prospectively analyzed the incidence of CNS tumors in all age groups [20]. They found 580 cases of primary CNS neoplasms and 78 cases of metastatic tumors. According to their data primary brain tumors most commonly presented in middle age men as 36% of their patients presented between 19 to 40 years of age. We also found middle age group as the most favorable for the occurrence of glial tumors. Astrocytomas were the most common among all CNS tumors in their study making up 30% of the primary tumors of CNS with high grade gliomas outnumbering low grade gliomas correlating positively with the results of our study.

Liaquat National Hospital is one of the largest tertiary care centers in the country which provides comprehensive neurosurgical facilities and therefore has a high influx of patients referred for neurosurgical problems. Patient population in neurosurgical unit represents both rural and urban sector of the province which reflects high rate of CNS tumors especially in urban areas of the country. The comparative analysis of the results of our study with that of local data is compared in Table 4.

**Table 4:** Comparative analysis with local data

**Comparative studies**	**Astrocytoma**	**Oligodendroglioma**	**Ependymoma**	**Mixed Oligoastrocytoma**
**Ahmed Z et al. 2001, Karachi, Pakistan **[19]	**71.4%**	**15%**	**9.30%**	**3.20%**
**Butt MA et al. 2005, Lahore, Pakistan **[16]	**82.80%**	**4.80%**	**4.80%**	**2.40%**
**Ayaz B et al. 2011, Karachi, Pakistan **[18]	**75%**	**12.50%**	**0.09%**	**0.03%**
**Jalali R et al. 2008, India **[20]	**75.40%**	**0.06%**	**13.60%**	**0%**
**Current study**	**51.60%**	**23%**	**7.90%**	**15.90%**

Our study showed some interesting findings which seems to be different from data generally available from other parts of the country, one of which is a higher frequency of oligodendroglial and oligoastrocytic tumors, which correlates with the newer statistics of the western world which show a frequency upto 25% for oligodendrogliomas. This trend towards a higher incidence of oligodendroglial tumors may represent a better understanding of tumor morphology after recognition of genetic signatures of oligodendroglial tumors or a change in the risk factors in the population. This may have serious implications on management as glial tumors with oligodendroglial component are more radiosensitive and they may have a better response to targeted chemotherapy to 1p19q co-deletions [21].

Most of studies done so far in Pakistan evaluating the frequency of CNS tumors were based on pathological data and they evaluated the frequency of all CNS tumors including metastatic tumors to brain and spinal cord. They consequently overemphasized that the frequency of metastatic tumors to brain in Pakistan are far less than compared to western population [8,9,14,15]. This may not be entirely true as brain surgery or even biopsy is not indicated in widely metastatic disease as the disease prognosis is extremely poor [22]. The tumors which most commonly metastasize to brain are carcinomas of lung, breast kidney and colon. The diagnosis of brain metastasis in known cases of these tumors can be made with the help of neuroimaging and serum biomarkers without the need of brain biopsy in most of the times.

In view of differences in management, molecular testing should be performed on every case of diffuse low grade cortical gliomas in adults in order to differentiate between diffuse astrocytomas and oligodendrogliomas. However in resource limited countries like Pakistan, where molecular testing is not widely available, we recommend that molecular testing should atleast be performed in histologically diagnosed oligodendroglioma and in those cases where prominent features of either astrocytomas and oligodendrogliomas are not present or a suspicion of oligodendroglioma component is raised histologically. This is because in current day medical practice presence of 1p19q co-deletion positive oligodendroglial component has a huge impact on therapeutic and diagnostic outcomes of patients.

Our data needs to be viewed with a few limitations. The data is based on a single institution and may not completely represent the profile of the entire population. Being a tertiary care centre also introduces confounding factors, such as bias in referred patterns. Another area of concern is that, being a laboratory based study, detailed clinical history including duration of symptoms and in a few cases neuroimaging was also not available for clinicopathologic correlation.

Despite a few limitations our data represents comprehensively the morphologic spectrum of glial tumors including grade characterization in our setup with age and sex distribution and has major implications on future research in this particular area.

## Conclusion

Astrocytomas are the most common glial tumors in our population with high grade IV astrocytoma (glioblastoma) being most common especially in older population, however the oligodendrogliomas and mixed oligoastrocytomas are seen more frequently in our population. Knowledge of these changing trends and patterns of glial tumor morphology and frequency can help in improvements and applications of newly emerging diagnostic and treatment modalities. Moreover further studies evaluating the genetic signatures of oligodendroglial tumors can help in identifying risk factors predisposing to these tumors in our population.

## Competing interests

The authors declare that they have no competing interests.

## Authors’ contributions

AAH: main author of manuscript, have made substantial contributions to conception, design and acquisition of data.NF: revising it critically for important intellectual content.BM: revising it critically for important intellectual content .MME: main author of manuscript, have made substantial contributions to conception, analysis and interpretation of data. AK: have given final approval of the version to be published. MK: have been involved in drafting the manuscript .All authors read and approved the final manuscript.
